# Rezidivcholesteatom nach Gehörgangsrekonstruktion: Widersprüchliche Befunde und differenzialdiagnostische Herausforderungen

**DOI:** 10.1007/s00106-024-01537-5

**Published:** 2024-12-06

**Authors:** F. Koller, C. Schmit, B. Henninger, N. Fischer, B. Hofauer, J. Schmutzhard

**Affiliations:** 1 Landeskrankenhaus (Univ.-Kliniken) Innsbruck, Innsbruck, Österreich; 2 Univ.-Klinik für Hals‑, Nasen- und Ohrenheilkunde, Medizinische Universität Innsbruck, Anichstraße 35, 6020 Innsbruck, Österreich; 3 Univ.-Klinik für Radiologie, Landeskrankenhaus (Univ.-Kliniken) Innsbruck, Innsbruck, Österreich

## Anamnese

Eine 48-jährige Patientin stellte sich mit neu aufgetretener Otorrhö bei drei Jahre zuvor erfolgter Entfernung eines ausgedehnten Cholesteatoms in der Hals-Nasen-Ohren-Klinik vor.

Die Patientin weist eine angeborene Gehörgangsatresie und Ohrmuschelaplasie auf, welche bereits im Kindesalter operativ rekonstruiert wurden. Das über 30 Jahre später diagnostizierte ausgedehnte Cholesteatom wurde mittels „Blind-Sac-Closure-Prozedur“ operativ versorgt. Postoperativ wurde die Patientin in engmaschige klinische und bildgebende Verlaufskontrollen eingebunden.

In den regelmäßig durchgeführten bildgebenden Kontrollen mittels Magnetresonanztomographie (MRT) zeigte sich zuletzt der Verdacht auf ein Rezidivcholesteatom im Bereich der Mittelohrhöhle. Aufgrund der konstanten Größenausdehnung des diffusionsgestörten Areals sowie der komplexen Vorgeschichte der Patientin hatte man sich zunächst auf eine abwartende Strategie geeinigt.

Zum Zeitpunkt der Vorstellung berichtet die Patientin über keinerlei sonstige Symptome. Insbesondere werden sonstige otologische Beschwerden wie Tinnitus, Schwindel, zunehmende Hörminderung oder Otalgie verneint. Anamnestisch bestand einen Monat zuvor, im Rahmen eines vom Allgemeinmediziner geäußerten Verdachts auf einen Infekt, das Gefühl „Wasser im Ohr“ zu haben. Trotz klinisch zum damaligen Zeitpunkt bestehendem reizlosem Lokalbefund erfolgte eine empirische antibiotische Therapie.

## Klinische Untersuchung

Ohrmikroskopisch zeigten sich rechtsseitig unauffällige anatomische Verhältnisse. Linksseitig präsentierte sich der blind endende Gehörgangssack ohne erkennbare entzündliche Veränderungen. Ein durchgeführtes Tonaudiogramm ergab rechtsseitig einen regelrechten altersentsprechenden Befund sowie linksseitig das Bild einer kombinierten Schwerhörigkeit mit einer Schallleitungskomponente von bis zu 60 dB. Abgesehen vom endoskopischen Befund einer posterioren Laryngitis zeigte sich der restliche HNO-Status unauffällig.

## Diagnostik und weiteres Prozedere

**Abb. 1:**
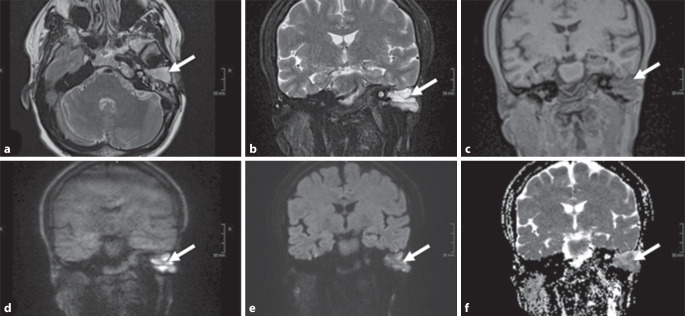
Rezentes MRT unter Verwendung des Cholesteatom-Protokolls. Der *weiße Pfeil* zeigt die Läsion, die wir als höchst verdächtig für ein Cholesteatom erachteten. Die T2w-Aufnahmen (**a** und **b**) zeigen eine starke Signalverstärkung, die in den T1w-Sequenzen (**c**) hypointens erscheint. Die diffusionsgewichtete Bildgebung (DWI) mit Non-EPI-HASTE (**d**) und EPI-DWI mit RESOLVE (**e**) ergibt eine starke Diffusionsrestriktion, die durch ein stark hypointenses Signal in der ADC (**f**) bestätigt wird

Die letzte bildgebende Diagnostik wurde mittels MRT nach Cholesteatom-Protokoll (Abb. 1) 11 Tage vor der aktuellen Vorstellung durchgeführt.

**Abb. 2:**
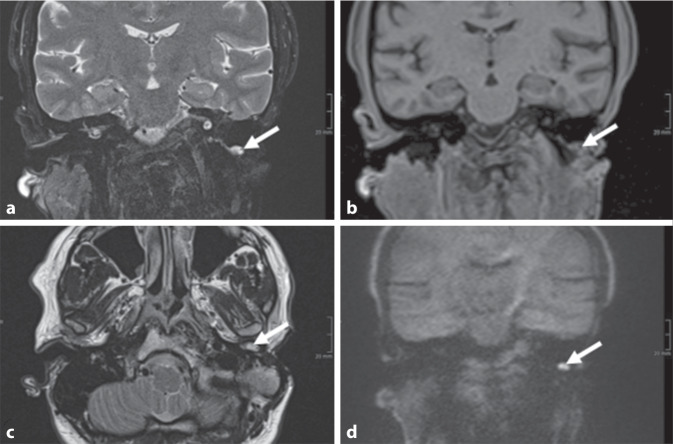
Initiale MRT unter Verwendung des Cholesteatom-Protokolls. Hier zeigt sich eine T2-hyperintense Läsion links (**a** koronales T2w-Bild mit Fettsättigung, **c** axiales T2w) dorsal des Kondylenfortsatzes, am Boden der Mittelohrhöhle. Die Läsion zeigte keine Signalverstärkung im T1w-Bild (**b**). In der Non-EPI-HASTE-DWI wurde eine deutliche Diffusionsrestriktion nachgewiesen (**d**). Daher wurde die Läsion als Cholesteatom eingeschätzt

Exakt ein Jahr zuvor zeigte sich bildgebend der Verdacht auf ein größenkonstantes Rezidivcholesteatom am Boden der Mittelohrhöhle (Abb. 2).

**Abb. 3:**
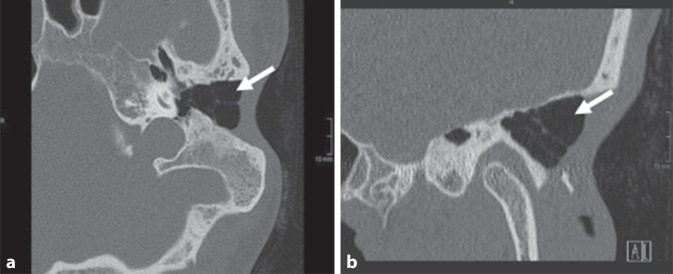
Felsenbein-CT: Die in der kürzlich durchgeführten MRT-Untersuchung als rezidivcholesteatomverdächtige Läsion beschriebene Raumforderung stellt sich sowohl in axialer (**a**) wie auch in koronaler (**b**) Ebene als ein luftgefüllter Hohlraum dar (*weißer Pfeil*)

Zur weiteren Abklärung der deutlich ersichtlichen Befundprogredienz erfolgte eine Felsenbein-CT (Abb. 3).

Entsprechend der Verdachtsdiagnose wurde eine zeitnahe operative Revision durchgeführt.

## Wie lautet Ihre Diagnose?

**Diagnose:** Verdacht auf Rezidivcholesteatom bei Zustand nach „Blind-Sac-Closure-Prozedur“

## Therapie und Verlauf

Entsprechend der Verdachtsdiagnose erfolgte eine operative Ohrrevision auf der linken Seite. Intraoperativ konnte der in der durchgeführten Magnetresonanztomographie geäußerte Verdacht auf ein ausgedehntes Cholesteatomrezidiv nicht bestätigt werden, es handelte sich somit um einen falsch-positiven Befund. Analog zur präoperativ durchgeführten CT zeigte sich die Mastoidektomiehöhle luftgefüllt und ohne ersichtliche Cholesteatommassen. Ein kleines Cholesteatom, entsprechend dem in Abb. 2 dargestellten Befund, wurde im Rahmen desselben operativen Eingriffs komplikationslos entfernt. Die postoperativ durchgeführte Knochenleitung zeigte einen identischen Befund wie präoperativ.

Die Patientin wurde postoperativ erneut in engmaschige klinische und bildgebende Kontrollen eingebunden.

## Diskussion

Das Cholesteatom, auch als chronische Otitis media epitympanalis bekannt, stellt eine Ansammlung von verhornendem Plattenepithel in der Paukenhöhle und/oder dem Mastoid dar, die sich klinisch ähnlich wie ein gutartiger Tumor verhält [1, 2].

Die Behandlung der chronischen Otitis media epitympanalis ist grundsätzlich chirurgisch [2, 3]. Hierbei wird zwischen der „Canal-Wall-down-Technik“, bei der eine Radikalhöhle angelegt wird, und der „Canal-Wall-up-Technik“, die den Erhalt oder die Rekonstruktion der hinteren Gehörgangswand anstrebt, unterschieden [2]. Aufgrund einer Rezidivrate von bis zu 27 % [4] und der häufig eingeschränkten postoperativen klinischen Beurteilbarkeit gewinnt eine adäquate Bildgebung zunehmend an Bedeutung bei der Detektion von Rezidivcholesteatomen [5, 6]. Die häufig angewendete Computertomographie bietet diesbezüglich jedoch lediglich eine sehr eingeschränkte Sensitivität und Spezifität und vermag nicht verlässlich zwischen Rezidiven und postoperativen Veränderungen unterscheiden [5]. Die zunehmend eingesetzte diffusionsgewichtete Magnetresonanztomographie (DWI-MRT) stellt, mit einer Sensitivität von 77,3 % und einer Spezifität von 72,7 %, eine effektive, nichtinvasive Methode zur Diagnostik von Rezidivcholesteatomen dar [5]. Durch die Einbringung des Beobachtervertrauens des erfahrenen befundenden Radiologen steigt die Zuverlässigkeit der DWI-MRT signifikant [5]. Die in der DWI-MRT beobachtete hohe Signalintensität resultiert am ehesten aus der eingeschränkten Diffusion von Wassermolekülen in den Keratinschuppen [5].

In dem vorgestellten Fall zeigte sich im Bereich der Mastoidektomiehöhle in der durchgeführten DWI-Sequenz eine ausgeprägte Diffusionsstörung, entsprechend der Vorgeschichte der Patientin suggestiv für ein ausgeprägtes Rezidivcholesteatom. Dies bestätigte sich jedoch in der kurz im Anschluss durchgeführten Computertomographie des Felsenbeins nicht. Hier zeigte sich die entsprechende Höhle luftgefüllt, ohne Hinweis auf ein Rezidiv. Auch im Rahmen der operativen Revision bestätigte sich der in der DWI-Sequenz geäußerte Verdacht nicht. Es zeigte sich lediglich das aus den vorherigen Bildgebungen bereits vorbekannte, zuletzt größenkonstante Cholesteatom am Boden der Mittelohrhöhle. Dieses konnte komplikationslos entfernt werden.

Trotz hoher Sensitivität und Spezifität der DWI-Sequenz in der Diagnostik von Rezidivcholesteatomen sind falsch-positive Befunde durch eine Fehlinterpretation von Diffusionsstörungen anderer Genese, wie beispielsweise durch Zerumenansammlung, Hämorrhagie oder entzündliches Gewebe möglich [5]. In dem vorgestellten Fall ist differenzialdiagnostisch, trotz weitestgehend unauffälliger Klinik und laborchemischen Parametern, am ehesten von einer zum Zeitpunkt der durchgeführten Bildgebung bestehenden eitrigen Entzündung der Mittelohrhöhle mit einhergehender Diffusionsstörung auszugehen. Die in weiterer Folge eingeleitete empirische antibiotische Therapie könnte die Rückbildung der entzündlichen Veränderungen erklären.

Letztlich bleibt die genaue Ursache der Diffusionsstörung aufgrund der spontanen Ausheilung zum Zeitpunkt der operativen Revision ungeklärt.

## Fazit für die Praxis

- In der Nachsorge des Cholesteatoms sind neben der klinischen Untersuchung bildgebende Verfahren von besonderer Bedeutung.
- Der DWI-MRT kommt mit einer Sensitivität von 77,3 % und einer Spezifität von 72,7 % eine zentrale Rolle in der Detektion von Rezidivcholesteatomen zu.
- Eine Diskrepanz zwischen DWI-MRT-Befunden und intraoperativen oder CT-Ergebnissen erfordert eine differenzierte Betrachtung und kann auf alternative pathologische Prozesse hindeuten.
- Eine umfassende interdisziplinäre Zusammenarbeit und eine Kombination aus klinischer Beurteilung, Bildgebung und operativer Revision sind entscheidend für die adäquate Diagnose und Behandlung von Rezidivcholesteatomen
